# What decision-making models can tell us about tactile remapping

**DOI:** 10.1186/1471-2202-12-S1-P311

**Published:** 2011-07-18

**Authors:** Larissa Albantakis, Krista E Overvliet, Elena Azañón, Gustavo Deco, Salvador Soto-Faraco

**Affiliations:** 1 Department of Information and Communication Technologies, Universitat Pompeu Fabra, Barcelona, 08003, Spain; 2 Departament de Psicologia Bàsica, Universitat de Barcelona, 08035 Barcelona, Spain; 3 Laboratory of Experimental Psychology, University of Leuven, 3000 Leuven, Belgium; 4 Institució Catalana de la Recerca i Estudis Avançats (ICREA), Universitat Pompeu Fabra, Barcelona, 08010, Spain

## 

To locate an object by touch, for example when fumbling around in the dark, or to react to a tactile event on the skin, somatosensory information must be quickly combined with the current position of the hand relative to the rest of the body. Not much is know yet about the time course of this integration between touch and body posture (e.g., proprioception), called tactile remapping. A tactile localization task in which there is a spatial conflict between the somatotopic and the external spatial representations can provide some constraints on the timing of the proprioceptive signal (necessary for the remapping) relative to the tactile input. Here, we construe this localization task as a perceptual discrimination problem. By making use of established models of decision-making, we aim to shed light on the internal processes during tactile remapping.

In the experiment, participants had to make a speeded saccade towards a brief somatosensory stimulus applied to the finger of one hand, while their arms were either crossed or uncrossed about the body midline. The eye movement trajectories were recorded and revealed that especially in the crossed condition fast saccades occasionally started toward the incorrect (anatomically congruent) side and were corrected in-flight, resulting in a turn-around saccade (about 250 ms after tactile onset, which might reflect the completion of tactile remapping). Similar curved traces have been observed recently in a random-dot motion (RDM) discrimination task were subjects had to report their decision by moving a handle to a left or right target [1]. While in the RDM task the curved traces were interpreted as a change of mind, direction-reversal here is thought to be induced by the (delayed) proprioceptive signal. With crossed hands, the current body posture needs to be taken into account and the representation of touch changes through time due to the remapping [2]. In the uncrossed posture remapping is trivial as the anatomical and external reference frames are aligned.

In the framework of decision-making and in line with [1], we treat the initial direction of a saccade as a first choice, caused by crossing a decision-threshold. Accordingly, we apply two computational models able to account for changes of mind, a simple drift-diffusion model [1] and a biophysically-realistic nonlinear attractor model [3], to fit experimental reaction-time distributions and performance. In the models, turn-around saccades originate from a second threshold crossing if the accumulated evidence of the initially losing alternative overtakes the first choice. Remapping only the outcome of the decision to a different motor response in the crossed-hand condition proved insufficient to explain the differences in reaction times and performance to the uncrossed case. This suggests that internal differences between the two conditions are already present prior to the decision-making stage. From the modeling perspective the proprioceptive signal thus changes the input (evidence) for deciding on a saccade to the left or right. Allowing only the model inputs to vary between crossed and uncrossed conditions, we gain predictions on the relative size and timing of the internal proprioceptive signal for tactile remapping.

## References

[B1] Resulaj A Kiani R Wolpert DM Shadlen MN Changes of mind in decision-making Nature 2009 461 7261 263 266 19693010 10.1038/nature08275 PMC2875179

[B2] Azañón E Soto-Faraco S Changing reference frames during the encoding of tactile events Curr Biol 2008 18 14 1044 1049 10.1016/j.cub.2008.06.045 18619841

[B3] Wang XJ Probabilistic decision making by slow reverberation in cortical circuits Neuron 2002 36 955 968 10.1016/S0896-6273(02)01092-9 12467598

